# Newer long-acting insulin prescriptions for patients with type 2 diabetes: prevalence and practice variation in a retrospective cohort study

**DOI:** 10.3399/BJGP.2021.0581

**Published:** 2022-05-24

**Authors:** Marloes Dankers, Karin Hek, Marjorie Nelissen-Vrancken, Sebastiaan T Houweling, Aukje Mantel-Teeuwisse, Liset van Dijk

**Affiliations:** Dutch Institute for Rational Use of Medicine, Utrecht, and PhD student, Department of PharmacoTherapy, Epidemiology & Economics, Groningen Research Institute of Pharmacy, Faculty of Science and Engineering, University of Groningen, Groningen.; Nivel, Netherlands Institute for Health Services Research, Utrecht.; Dutch Institute for Rational Use of Medicine, Utrecht.; General Practice Sleeuwijk, Sleeuwijk.; Division of Pharmacoepidemiology & Clinical Pharmacology, Utrecht Institute for Pharmaceutical Sciences, Utrecht University, Utrecht.; Nivel, Netherlands Institute for Health Services Research, Utrecht, and honorary professor, pharmacy health services research, Department of PharmacoTherapy, Epidemiology & Economics, Groningen Research Institute of Pharmacy, Faculty of Science and Engineering, University of Groningen, Groningen.

**Keywords:** diabetes mellitus, insulin, primary health care, practice variation

## Abstract

**Background:**

Little is known about prescription patterns of expensive non-recommended newer long-acting insulins (glargine 300 U/mL and degludec) for patients with type 2 diabetes mellitus (T2DM).

**Aim:**

To identify practice variation in, and practice- and patient-related characteristics associated with, the prescription of newer long-acting insulins to patients with T2DM in primary care.

**Design and setting:**

A retrospective cohort study in Dutch general practices (Nivel Primary Care Database).

**Method:**

A first prescription for intermediate or long-acting insulins in 2018 was identified in patients aged ≥40 years using other T2DM drugs. Per practice, the median percentage and interquartile range (IQR) of patients with newer insulin prescriptions were calculated. Multilevel logistic regression models were constructed to calculate intraclass correlation coefficients (ICCs) and quantify the association of patient and practice characteristics with prescriptions for newer insulins (odds ratios [ORs] and 95% confidence intervals [CIs]).

**Results:**

In total, 7757 patients with prescriptions for intermediate or long-acting insulins from 282 general practices were identified. A median percentage of 21.2% (IQR 12.5–36.4%) of all patients prescribed intermediate or long-acting insulins per practice received a prescription for newer insulins. After multilevel modelling, the ICC decreased from 20% to 19%. Female sex (OR 0.77, 95% CI = 0.69 to 0.87), age ≥86 years compared with 40–55 years (OR 0.22, 95% CI = 0.15 to 0.34), prescriptions for metformin (OR 0.66, 95% CI = 0.53 to 0.82), sulfonylurea (OR 0.58, 95% CI = 0.51 to 0.66), or other newer T2DM drugs (OR 3.10, 95% CI = 2.63 to 3.66), and dispensing practices (OR 1.78, 95% CI = 1.03 to 3.10) were associated with the prescription of newer insulins.

**Conclusion:**

The inter-practice variation in the prescription of newer insulins is large and could only be partially explained by patient- and practice-related differences. This indicates substantial opportunities for improvement.

## INTRODUCTION

New medicines are often expensive and have a risk–benefit ratio that has not been fully elucidated yet.^1^^,^^2^ Therefore, clinical guidelines usually do not recommend their use, especially if less expensive and evidence-based alternatives are available.^3^ This is also reflected in the most current type 2 diabetes mellitus (T2DM) guidelines, which do not recommend the use of insulin glargine 300 U/mL and insulin degludec.^4^^–^^6^ These two most recently introduced long-acting insulins, further referred to as ‘newer insulins’, gained market access in 2013 and 2015, respectively.

In the Netherlands, the majority of insulins for T2DM are prescribed in primary care.^7^ The guideline of the Dutch College of General Practitioners *NHG-Standaard diabetes mellitus type 2* advises against the use of the newer insulins, for reasons of non-evidence-based advantages compared with other intermediate and long-acting insulins.^5^ In addition, insulin degludec has an unknown long-term safety and is more expensive than other insulins. In the Netherlands, insulin glargine 300 U/mL is also slightly more expensive than other insulins, and safety concerns about high-strength concentration and risk of dose error exist.

The Dutch guideline considers NPH-insulin as the first choice with insulin glargine 100 U/mL and insulin detemir as potential alternatives in specific situations.^5^ Although adherence to guidelines is generally high among Dutch GPs,^8^^,^^9^ the popularity of insulin glargine 300 U/ml and insulin degludec in Dutch practice is rapidly increasing.^10^ However, little is known about patterns of newer insulin use in patients with T2DM and especially information on practice variation and practice- and patient-related characteristics associated with the prescription of newer long-acting insulins is lacking. In previous research, a number of patient and practice characteristics have been positively associated with the prescription of new medicines, including male sex, younger age, and practice location.^11^^,^^12^ Whether these factors also apply to the prescription of newer insulins is unknown.

To stimulate better quality of care and prevent increasing expenditure on insulins for patients with T2DM, insight into the prescription patterns of non-recommended newer insulins is warranted. This study therefore aimed to identify practice variation in, and practice and patient characteristics associated with, the prescription of newer insulins to patients with T2DM in primary care, 3–5 years after their introduction.

**Table:** How this fits in

Newer long-acting insulins are not recommended for the treatment of patients with type 2 diabetes according to the Dutch guideline *NHG-Standaard diabetes mellitus type 2.* However, this study shows that approximately a quarter of all patients with type 2 diabetes prescribed intermediate or long-acting insulins received a prescription for one of the newer insulins. Large variation among general practices existed, even after correction for differences at the patient and practice level. This indicates opportunities for quality improvement of the pharmaceutical treatment of patients with type 2 diabetes.

## METHOD

### Study setting and participants

Data from the Nivel Primary Care Database (Nivel-PCD) were used. Nivel-PCD collects data from routine electronic health records from a dynamic sample of approximately 500 general practices in the Netherlands (roughly 10% of the Dutch population).

Data include information on patient characteristics, consultations, morbidity, prescriptions, lab test results, and the patient’s main diabetes practitioner (primary or secondary care provider). The age and sex distribution of listed patients is representative of the general Dutch population.^13^

All patients with one or more prescriptions for intermediate-acting insulins (Anatomical Therapeutic Chemical Classification system [ATC code] A10AC) or long-acting insulins (A10AE) in 2018 were included. To distinguish between T1DM and T2DM, only patients using insulins, aged ≥40, and using one or more other blood glucose-lowering drugs were included.^14^

Insulin-naive patients were defined as having no prescription for any insulin (A10A) in 2017. Prescriptions for insulin glargine 100 U/ml and insulin glargine 300 U/ml were distinguished based on unique product codes. Prescriptions for insulin glargine with unknown product codes were excluded from further analysis (*n* = 47).

### Determinants

#### Patient characteristics

Age, sex, the number of chronic diseases, duration of T2DM (based on date of first diagnosis), and prescriptions for blood glucose lowering drugs other than insulin at any time in 2018 were included as patient characteristics.

Age was divided into four categories (40–55 years; 56–70 years; 71–85 years; and ≥86 years). T2DM duration was divided into six categories (0–5 years; 6–10 years; 11–15 years; 16–20 years; ≥21 years; and unknown). As a result of inaccurate recording of the year of diagnosis for a subset of patients (for example, the year of diagnosis was ‘01-01-1900’), duration was considered unknown if age at diagnosis was <40 years.

In order to evaluate comorbidities, a selection of 29 chronic diseases was made, using constructed disease episodes of recorded morbidity data from the electronic health records.^15^^,^^16^ The number of chronic diseases was divided into three categories (0–1, 2–4, and ≥5).^17^

#### Practice characteristics

Practice type (that is, solo, duo, and group) was analysed. Missing values (*n* = 19) were considered as a distinct category ‘unknown’. In addition, dispensing practices were distinguished from non-dispensing practices, with practices with unknown status (*n* = 12) being considered as non-dispensing. This was done because the vast majority of practices in the Netherlands are non-dispensing and so it is unlikely that a dispensing status would not be recorded accurately.

The socioeconomic status (SES) of the location of the practice (developed by the Netherlands Institute for Social Research),^18^ the percentage of patients aged ≥70 years, and practice size were divided in tertiles.^17^ The degree of urbanisation of practice locations was divided into five categories.

### Analysis

The number of patients with a prescription for an intermediate or long-acting insulin in 2018 was established for the entire cohort and for insulin-naive patients. The percentage of patients with a prescription for a newer insulin (insulin glargine 300 U/ml or insulin degludec) compared with all patients with an intermediate or long-acting insulin per practice was also analysed and the median percentage per practice and interquartile range (IQR) calculated.

Multivariate logistic regression analyses were performed on the entire cohort to assess the association of patient and practice characteristics with prescriptions for newer long-acting insulins (Stata SE version 16.1). To examine inter-practice variation, multilevel models with patients (level 1) clustered within general practices (level 2) was constructed, using a random-effects model.

An empty model (model 1) with only the dependent variable (patients receiving a prescription for a newer insulin) was constructed to establish the a priori chance of a patient receiving a prescription for a newer insulin. In model 2, all patient characteristics were added. Model 3 contained all patient characteristics (level 1) and practice characteristics (level 2).

All variables were included simultaneously, so all independent variables in the multilevel analysis were mutually adjusted for, thereby minimising the risk of confounding by these factors. The likelihood-ratio test was performed to establish the ‘fit’ of both models. Odds ratios (ORs), 95% confidence intervals (95% CIs), and *P*-values were calculated to indicate the association between prescriptions for newer insulins and the dependent variables. Intraclass correlation coefficients (ICC) were calculated to indicate the relative contribution of variation at the practice level (level 2) to the total variation. Missing values were considered as an unknown category in the multivariate analysis.

As the patient’s main diabetes practitioner was unknown for a subset of patients and prescriptions from secondary care providers could have contributed to the results, an additional multilevel analysis with only those patients with the GP as the main responsible treating physician for T2DM was also performed.

## RESULTS

### Baseline characteristics

A total of 7757 patients from 282 general practices received a prescription for an intermediate or long-acting insulin (Tables 1 and 2). There were 1159 patients (14.9%) who were insulin-naive. Insulin-naive patients were younger and had a shorter duration of T2DM than the overall population. The patient’s main diabetes practitioner was known for 4529/7757 (58.4%) of all patients, 4032/7757 (52.0%) for the GP, and 497/7757 (6.4%) for the specialist.

**Table 1.** Baseline characteristics of patients

**Characteristic**	**All patients (*n*= 7757)**	**Insulin-naive patients (*n*= 1159)**
**Sex, *n* (%)**		
Male	4268 (55.0)	647 (55.8)
Female	3489 (45.0)	512 (44.2)

**Age, years, mean (SD)**	67.4 (11.0)	65.8 (12.0)

**Age, in categories, *n* (%)**		
40–55 years	1221 (15.7)	258 (22.3)
56–70 years	3324 (42.9)	464 (40.0)
71–85 years	2859 (36.9)	376 (32.4)
≥86 years	353 (4.6)	61 (5.3)

**Number of chronic diseases, mean (SD)**	3.7 (1.8)	3.5 (1.8)

**Chronic diseases, in categories**		
0–1 diseases	665 (8.6)	127 (11.0)
2–4 diseases	4882 (62.9)	746 (64.4)
≥5 diseases	2210 (28.5)	286 (24.7)

**Duration of T2DM, years, mean (SD)**	13.1 (6.1)	9.9 (5.9)

**Duration of T2DM, in categories**		
0–5 years	708 (9.1)	256 (22.1)
6–10 years	1624 (20.9)	309 (26.7)
11–15 years	2217 (28.6)	308 (26.6)
16–20 years	1465 (18.9)	129 (11.1)
≥21 years	790 (10.2)	41 (3.5)
Unknown	953 (12.3)	116 (10.0)

**Number of blood glucose lowering drugs, mean (SD)**	1.5 (0.62)	1.9 (0.73)

**Drug**		
Metformin	7098 (91.5)	1033 (89.1)
Sulfonylurea	3502 (45.1)	863 (74.5)
Dipeptidyl peptidase-4 inhibitors	286 (3.7)	167 (14.4)
Glucagon-like peptide-1 agonists	399 (5.1)	74 (6.4)
Sodium-glucose co-transporter-2 inhibitors	306 (3.9)	63 (5.4)
Acarbose	15 (0.2)	2 (0.2)
Meglitinides	13 (0.2)	1 (0.1)
Thiazolidinediones	31 (0.4)	8 (0.7)

*SD = standard deviation. T2DM = type 2 diabetes mellitus.*

**Table 2.** Baseline characteristics of practices

**Characteristic**	**All patients**		**Insulin-naive patients**	
	
	**Practices (*n*= 282)**	**Patients (*n*= 7757)**	**Practices (*n*= 262)**	**Patients (*n*= 1159)**
**Type of practice, *n* (%)**				
Single-handed practice	67 (23.8)	1534 (19.8)	61 (23.3)	209 (18.0)
Duo practices	95 (33.7)	1939 (25.0)	86 (32.8)	278 (24.0)
Group practices	101 (35.8)	3852 (49.7)	97 (37.0)	598 (51.6)
Unknown	19 (6.7)	432 (5.6)	18 (6.9)	74 (6.4)

**Dispensing practice,^a^ *n* (%)**	8 (2.8)	185 (2.4)	7 (2.7)	24 (2.1)

**Practice size, mean number of patients (SD)**	—	3902.5 (2402.0)	—	3964.7 (2419.7)

**Practice size groups,^b^ *n* (%)**				
Small	94 (33.3)	1592 (20.5)	78 (29.8)	206 (17.8)
Medium	94 (33.3)	1948 (25.1)	90 (34.4)	307 (26.5)
Large	94 (33.3)	4217 (54.4)	94 (35.9)	646 (55.7)

**Degree of urbanisation (location of practice), *n* (%)**				
Very strong	71 (25.2)	2184 (28.2)	69 (26.3)	389 (33.6)
Strong	69 (24.5)	1864 (24.0)	62 (23.7)	267 (23.0)
Moderate	64 (22.7)	1799 (23.2)	60 (22.9)	244 (21.1)
Little	44 (15.6)	1143 (14.7)	41 (15.6)	172 (14.8)
Not	34 (12.1)	767 (9.9)	30 (11.5)	87 (7.5)

**SES (location of practice), *n* (%)**				
Low	94 (33.3)	2944 (38.0)	92 (35.1)	420 (36.2)
Moderate	94 (33.3)	2618 (33.8)	85 (32.4)	387 (33.4)
High	94 (33.3)	2195 (28.3)	85 (32.4)	352 (30.4)

**% aged ≥70 years^c^**				
Low	94 (33.3)	2382 (30.7)	87 (33.2)	376 (32.4)
Moderate	94 (33.3)	2483 (32.0)	85 (32.4)	387 (33.4)
High	94 (33.3)	2892 (37.3)	90 (34.4)	396 (34.2)

a *Status was unknown for 12 practices.*

b *Small 1337–2599 patients; medium 2601–3782 patients; large 3828–16 923 patients.*

c *Low:* <*12.6%; medium 12.7–16.2%; high* >*16.2%. SD = standard deviation. SES = socioeconomic status.*

### Patients with newer insulins and practice variation

In total, 25.6% (1983/7757) of all patients received a prescription for one of the newer insulins (14.0% degludec and 11.5% glargine 300 U/mL) (Table 3). The proportion of patients with a prescription for newer insulins was comparable between insulin-naive and non-naive patients.

**Figure 1.**
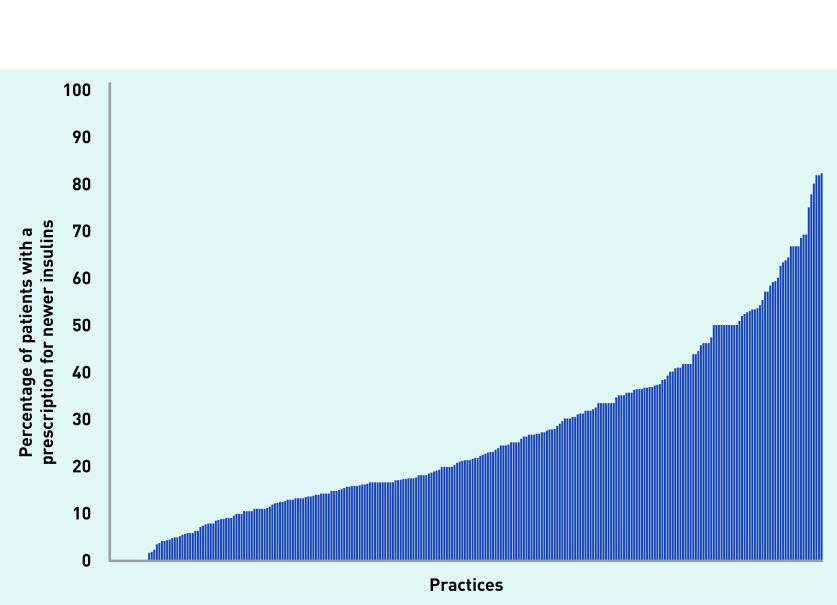
***Percentage of patients with a prescription for a newer insulin relative to all prescriptions for intermediate or long-acting insulins.****^a^* *^a^****The* x *-axis shows the practices; the* y *-axis represents the percentage of patients with prescriptions for newer insulins. Each bar represents one practice. Median 21.2%, interquartile range 12.5–36.4%.***

The median percentage of patients with prescriptions per practice for newer insulins compared with all intermediate and long-acting insulins was 21.2% (IQR 12.5–36.4%) (Figure 1), showing considerable practice variation. Differences in prescribing patterns for these two newer insulins can be found in Supplementary Figures S1 and S2 for glargine 300 U/mL and degludec, respectively.

**Table 3.** Number of patients with a prescription for intermediate and long-acting insulins

**Type of insulin**	**All patients, *n* (%) (*n*= 7757)**	**Insulin-naive patients, *n* (%) (*n*= 1159)**
**Newer insulins**	1983 (25.6)	282 (24.3)
Glargine 300 U/ml	895 (11.5)	120 (10.4)
Degludec	1088 (14.0)	162 (14.0)

**Other insulins**	5774 (74.4)	877 (75.7)
NPH-insulin	1330 (17.1)	303 (26.1)
Glargine 100 U/ml	3516 (45.3)	501 (43.2)
Detemir	928 (12.0)	73 (6.3)

### Determinants of patients with prescriptions for newer insulins

The a priori odds for a prescription of a newer insulin was 29% (OR empty model, Table 4). The corresponding ICC was 95% CI = 0.17 to 0.25), meaning that 20% of the observed variability could be attributed to differences between practices. There was only a minor decrease in the intraclass correlation coefficient I(CC) after including both patient and practice characteristics (from 0.20 to 0.19), suggesting that most of the practice variation could not be explained by the factors included in the model (data not shown).

**Table 4.** Results of multivariate analysis

	**Empty model**		**Model 1**		**Model 2**	
		
	**OR**	**95% CI**	**OR**	**95% CI**	**OR**	**95% CI**
**Overall outcome**	0.29^a^	0.26 to 0.33	—	—	—	—

**Female sex**	—	—	0.77^a^	0.69 to 0.87	0.77^a^	0.69 to 0.87

**Age, years**						
40–55 years	Reference	—	—	—	—	—
56–70 years	—	—	0.70^a^	0.58 to 0.85	0.70^a^	0.58 to 0.85
71–85 years	—	—	0.38^a^	0.31 to 0.47	0.38^a^	0.30 to 0.47
≥86 years	—	—	0.23^a^	0.15 to 0.34	0.22^a^	0.15 to 0.34

**Chronic disease**						
0–1 chronic diseases	Reference	—	—	—	—	—
2–4 chronic diseases	—	—	1.15	0.93 to 1.43	1.15	0.93 to 1.43
≥5 chronic diseases	—	—	1.27^b^	1.00 to 1.62	1.27	1.00 to 1.62

**Years since T2DM diagnosis**						
0–5 years T2DM	Reference	—	—	—	—	—
6–10 years T2DM	—	—	1.10	0.88 to 1.38	1.11	0.89 to 1.39
11–15 years T2DM	—	—	0.98	0.78 to 1.23	0.99	0.79 to 1.24
16–20 years T2DM	—	—	0.94	0.73 to 1.20	0.94	0.74 to 1.21
≥21 years T2DM	—	—	1.24	0.93 to 1.64	1.24	0.93 to 1.64
Unknown^c^	—	—	—	—	—	—

**Prescription for metformin**	—	—	0.66^a^	0.53 to 0.82	0.66^a^	0.53 to 0.82

**Prescription for sulfonylurea**	—	—	0.58^a^	0.51 to 0.66	0.58^a^	0.51 to 0.66

**Prescription for DPP4 inhibitors, GLP1 agonists, or SGLT2 inhibitors**	—	—	3.12^a^	2.65 to 3.68	3.10^a^	2.63 to 3.66

**Type of practice**						
Single-handed practice	Reference	—	—	—	—	—
Duo practices	—	—	—	—	0.87	0.60 to 1.25
Group practices	—	—	—	—	0.91	0.61 to 1.37
Unknown^c^	—	—	—	—	—	—

**Dispensing practice**	—	—	—	—	1.78^a^	1.03 to 3.10

**Practice size**						
Small practice size	Reference	—	—	—	—	—
Medium practice size	—	—	—	—	1.49^a^	1.07 to 2.08
Large practice size	—	—	—	—	0.80	0.55 to 1.17

**Urbanisation**						
Very strong urbanisation	Reference	—	—	—	—	—
Strong urbanisation	—	—	—	—	1.26	0.86 to 1.84
Moderate urbanisation	—	—	—	—	1.01	0.69 to 1.46
Little urbanisation	—	—	—	—	0.87	0.56 to 1.33
No urbanisation	—	—	—	—	0.99	0.61 to 1.61

**SES**						
Low SES	Reference	—	—	—	—	—
Moderate SES	—	—	—	—	0.90	0.65 to 1.24
High SES	—	—	—	—	0.85	0.62 to 1.17

**Patients** ≥**70 years**						

Low number of patients ≥70 years	Reference	—	—	—	—	—

Moderate number of patients ≥70 years	—	—	—	—	1.26	0.91 to 1.74

High number of patients ≥70 years	—	—	—	—	1.49^a^	1.08 to 2.05

a *P*<*0.001;*

b *P = 0.05.*

c *Data not shown. CI = confidence interval. DPP4 = dipeptidyl peptidase-4. GLP1 = glucagon-like peptide-1. OR = odds ratio. SES = socioeconomic status. SGLT2 = sodium-glucose co-transporter-2. T2DM = type 2 diabetes.*

Some factors were associated with prescriptions for newer insulins. At the patient level, female sex (OR 0.77, 95% CI = 0.69 to 0.87), prescriptions for metformin (OR 0.66, 95% CI = 0.53 to 0.82) or sulfonylurea (OR 0.58, 95% CI = 0.51 to 0.66), and older age (OR 0.22, 95% CI = 0.15 to 0.34, for patients aged ≥86 years compared with patients aged 40–55 years) were inversely associated with prescriptions for newer insulins (Table 4).

Prescriptions for newer blood glucose lowering drugs other than insulins dipeptidyl peptidase-4 (DPP4) inhibitors, glucagon-like peptide-1 [GLP1] agonists, and sodium-glucose co-transporter-2 (SGLT2) inhibitors were the strongest predictor for a prescription for newer insulins: OR 3.10 (95% CI = 2.63 to 3.66). No significant association between prescriptions for newer insulins and the number of chronic diseases nor duration of T2DM was found (Table 4).

GPs in practices that dispense medication themselves prescribed newer insulins more often (OR 1.78, 95% CI = 1.03 to 3.10) (Table 4). Other practice characteristics were not consistently related to the prescriptions for newer insulins.

The multivariate analysis for patients with the GP as main practitioner consisted of 4032 patients in 213 practices. No relevant differences were observed compared with the main analyses (Supplementary Table S1).

## DISCUSSION

### Summary

In Dutch primary care, approximately a quarter of patients with T2DM with intermediate or long-acting insulins were prescribed the newer long-acting insulins insulin degludec or insulin glargine 300 U/ml, in spite of the current guideline advising other intermediate or long-acting insulins.^5^ Practice variation was extensive and largely remained after correction for patient and practice characteristics. Except for dispensing practices, no practice characteristics were unambiguously related to the prescription of newer insulins.

Male patients, younger patients, and patients with prescriptions for other newer blood glucose lowering agents (which do not have a prominent place in the Dutch guideline for T2DM) were more likely to receive a prescription for newer insulins. Patients with prescriptions for metformin or sulfonylurea were less likely to receive a prescription for newer insulins.

It therefore seems that guideline adherence in an earlier stage of T2DM treatment (that is, the prescription of metformin, sulfonylurea but not the other newer agents) is associated with guideline adherence in the later stages of T2DM management.

The major part of practice variation could not be explained. Therefore, other determinants are likely to have a significant influence on the prescription of newer insulins in primary care.

### Strengths and limitations

The main strength of this study is the use of a large and representative database from which medication prescriptions as well as patient and practice characteristics could be retrieved, thus avoiding selection bias, which might be inherent to population surveys.^13^ The large number of patients (*n* = 7757) and general practices (*n* = 282) contributed to stable and robust multilevel models.

There are, however, some limitations. As only a selection of patient and practice characteristics were included in this study, it is not known to which extent other factors (for example, the patient’s health status) contributed to the practice variation, and may confound the present results. Furthermore, it was not possible to distinguish insulin prescriptions by GPs from prescriptions by specialists. Although the main practitioner was identified for almost 60% of the included patients, it was not known whether this physician had indeed initiated insulin therapy. Nevertheless, as the analysis that was restricted to patients with the GP as main practitioner yielded similar results, a prominent role for differences between prescribers is unlikely. Finally, as diagnosis was not always recorded accurately, it was not possible to distinguish T2DM from T1DM based on recorded episodes, and the date of diagnosis was not always recorded accurately. However, as the analysis selected by age (≥40 years) and by prescriptions for other blood glucose lowering drugs than insulin, the possibility of including patients with T1DM was minimal.

### Comparison with existing literature

Most studies on insulin use focused on between-class variation (that is, comparison with the use of rapid-acting insulins and premixed insulins) rather than the in-between class variation.^19^^–^^25^

A recent analysis from the UK showed that prescription rates for long-acting insulins increased between 2003 and 2018, whereas prescription rates of NPH-insulin decreased. In the UK, of all patients who started degludec between 2013–2018, 38% started in 2018, indicating ongoing growth in uptake after its introduction in 2013.^26^

An analysis by Zhang *et al* of 5034 American patients with T2DM initiating insulins between 2014 and 2017 indicated that 6.5% used one of the newer insulins.^27^ Although there are substantial differences between the healthcare systems in the US and the Netherlands, the findings in the current study (which is more recent) of 26.0% might reflect increasing uptake over time. According to Zhang *et al*, users of newer insulins more often used more medications at baseline and were more likely to have experience with GLP1-agonists.^27^

Brunetti *et al* found that users of insulin degludec were more likely to have used other blood glucose lowering drugs before the initiation of insulin.^26^ The positive association with prescriptions for other newer blood glucose lowering agents was confirmed in the present study. Of note, in the current study prescriptions for metformin and sulfonylurea were found to be inversely related. Although in this study any association with the number of other medicines was not investigated, the lack of association with chronic diseases is not supportive for a strong association.

In the Netherlands, guideline adherence is generally high^8^^,^^9^ and it is therefore remarkable that a quarter of patients with intermediate or long-acting insulins were prescribed non-recommended newer insulins. The rapid uptake shows similarities with earlier investigations towards the uptake of the first generation of insulin analogues. After their market introduction, insulin glargine 100 U/ml and insulin detemir were rapidly adopted, resulting in increasing dispensing rates and healthcare costs.^23^^,^^25^^,^^28^^–^^30^ Significant regional variations in the use of the — at that moment — newer insulins were found.^23^^,^^28^

Patient-level factors, such as age and comorbidities, were thought to have a significant impact on the prescription rates,^28^ a suggestion that is only partially confirmed in the current investigation. The first-generation insulin analogues were more often adopted in internal medicine practices than in general practices. Owing to similarities in rapid uptake of the first and second generations of insulin analogues, the lessons learned from the uptake of first-generation insulin analogues will most likely also apply to the current situation.

The factors associated with the use of new medicines may vary between therapeutic areas.^1^ In line with the current findings, a recent analysis of the use of new medicines, irrespective of therapeutic area, in Switzerland found that male sex and younger age enhanced the probability of using new medicines, whereas the number of comorbidities had little impact.^12^ In contrast with the current findings, the practice location and proportion of older people in general practice have also been associated with the use of new medicines.^11^ Other factors, such as strong scientific commitment, high exposure to marketing, and extensive communication with colleagues, were also strongly associated with the use of (all) new medicines.^1^ As the current study could not investigate those determinants, it is not clear whether these factors also contribute to the prescriptions for newer insulins. As the majority of practice variation could not be explained by the determinants investigated in the current study, it is likely that external influences also affected the prescription of newer insulins.

### Implications for research and practice

The inter-practice variation in the prescription of newer insulins is large and could only be partially explained by patient- and practice-related differences. Therefore, more research into the reasons for non-adherence to guidelines is warranted, keeping in mind that physician beliefs and attitudes towards newer medicines may play a prominent role. This could lead to both relevant insights for guideline makers as well as directions for physician-centred interventions to stimulate qualitative and cost-effective prescribing behaviour.

In conclusion, in Dutch general practice, a substantial number of patients with T2DM received prescriptions for newer insulins, which are not recommended by the current guideline. After correcting for patient and practice characteristics, practice variation remained substantial. Other factors, such as physician beliefs and attitudes, are therefore likely to influence the prescription of newer insulins and there is a need for further research to examine this.

## Data

Access to data is subject to Nivel Primary Care Database governance codes. Requests for access to the data can be directed to directie@nivel.nl. Restrictions involve establishing a data sharing agreement and approval by the appropriate Nivel Primary Care Database governance bodies (privacy committee and steering committee). Data were anonymised and are accessible via https://doi.org/10.17026/dans-x9q-kz3n (DANS | Centre of expertise & repository for research data (knaw.nl).
